# Gene expression profiling discerns molecular pathways elicited by ligand signaling to enhance the specification of embryonic stem cells into skeletal muscle lineage

**DOI:** 10.1186/s13578-017-0150-x

**Published:** 2017-05-02

**Authors:** Katherine Dixon, Jihong Chen, Qiao Li

**Affiliations:** 1 Department of Cellular and Molecular Medicine, Faculty of Medicine, University of Ottawa, 451 Smyth Road, Room 2537, Ottawa, ON K1H 8M5 Canada; 2 Department of Pathology and Laboratory Medicine, Faculty of Medicine, University of Ottawa, Ottawa, ON Canada

**Keywords:** Gene regulation, Ligand, Nuclear receptors, Rexinoid, Skeletal myogenesis, Stem cell, Differentiation

## Abstract

Regulation of lineage specification and differentiation in embryonic stem (ES) cells can be achieved through the activation of endogenous signaling, an avenue for potential application in regenerative medicine. During vertebrate development, retinoic acid (RA) plays an important role in body axis elongation and mesoderm segmentation in that graded exposure to RA provides cells with positional identity and directs commitment to specific tissue lineages. Nevertheless, bexarotene, a clinically approved rexinoid, enhances the specification and differentiation of ES cells into skeletal myocytes more effectively than RA. Thus profiling the transcriptomes of ES cells differentiated with bexarotene or RA permits the identification of different genetic targets and signaling pathways that may contribute to the difference of bexarotene and RA in efficiency of myogenesis. Interestingly, bexarotene induces the early expression of a myogenic progenitor marker, *Meox1*, while the expression of many RA targets is also enhanced by bexarotene. Several signaling molecules involved in the progression of myogenic specification and commitment are differentially regulated by bexarotene and RA, suggesting that early targets of rexinoid allow the coordinated regulation of molecular events which leads to efficient myogenic differentiation in ES cells.

## Background

**Fig. 1:**
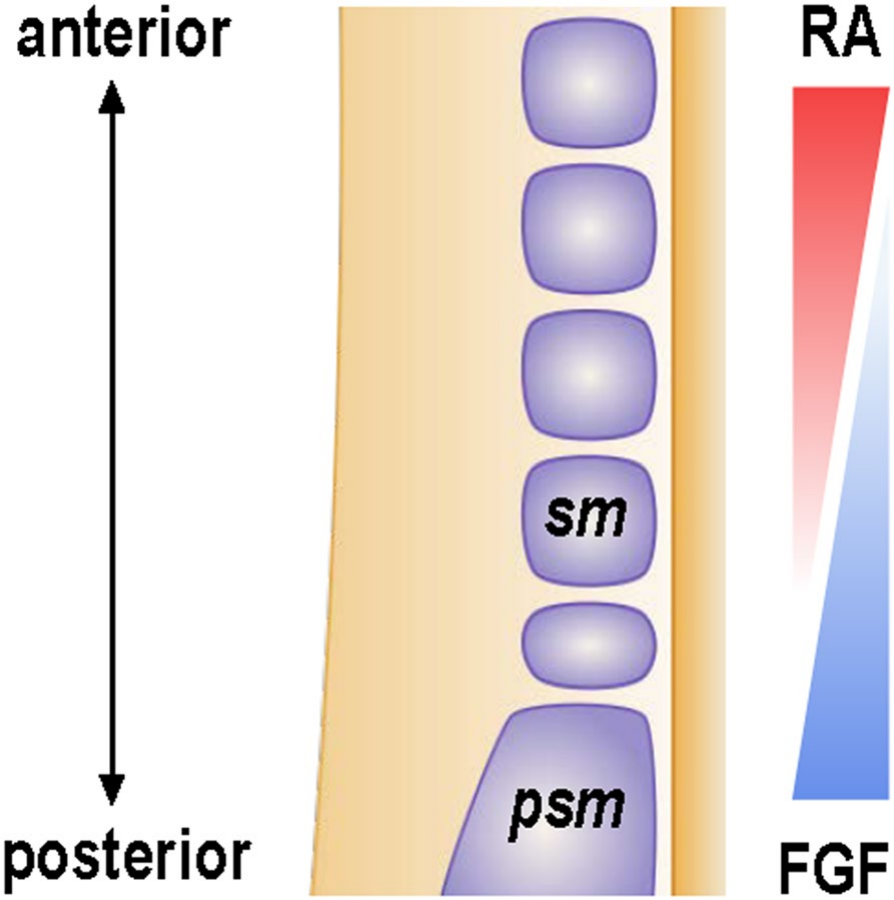
Schematic representation of RA and fibroblast growth factor (FGF) gradients in vertebrate embryos undergoing somitogenesis

During vertebrate embryogenesis, the progression of skeletal myogenesis is regulated by coordinated signaling pathways that induce the sequential expression of a range of key developmental transcription factors. Retinoic acid (RA), a derivate of vitamin A, is a morphogen that plays many important roles in vertebrate embryonic development [1, 2]. Prior to the specification of skeletal muscle lineage, anterior–posterior elongation of the body axis and accompanying segmentation of paraxial mesoderm are partly regulated by opposing gradients of RA and fibroblast growth factor (Fig. 1) [3]. At this stage of development, RA is synthesized in somitic mesoderm and diffuses towards each end of the embryo along the anterior–posterior axis, exerting spatial and temporal regulation of target genes [4]. In vitro, low concentrations of exogenous RA promote the differentiation of pluripotent stem cells into skeletal myocytes [5, 6]. While the generation of skeletal myocytes from pluripotent stem cells has beneficial applications in regenerative medicine, the efficiency of myogenic differentiation in RA-treated stem cells is relatively low and alternative approaches are therefore desirable [7–9]. Interestingly, bexarotene, a rexinoid used clinically as a treatment for cutaneous T-cell lymphoma, has the potential to be used as an alternative and more effective enhancer than RA during the in vitro differentiation of ES cells into skeletal myocytes [10, 11]. However, the differential effects of bexarotene and RA on genetic targets and transcriptional programs, especially those pertaining to skeletal myogenesis need to be understood and are therefore the focus of this article.

## Regulation of myogenic specification

The commitment and development of skeletal muscle lineage depends on the expression of the myogenic regulatory factors (MRFs), including Myf5, MyoD, myogenin and MRF4, a group of highly conserved basic helix-loop-helix (bHLH) transcription factors. Myf5 and MyoD are independently sufficient to induce myogenesis in non-muscle cell types such as fibroblasts [12, 13], and mice lacking both *Myf5* and *MyoD* completely lack skeletal muscle [14].

Myogenin and MRF4 are genetically downstream of Myf5 and MyoD. While myogenin is required for terminal differentiation of myoblasts into myotubes, loss of MRF4 results in only minor impairment in myotube formation [15, 16]. During embryonic development, the induction of *Myf5* and *MyoD* and the specification of myogenic progenitors in the dorsal somite, known as the dermomyotome, require coordination of signaling pathways activated by molecules secreted from adjacent tissues. In particular, sonic hedgehog (Shh) from the notochord, Wnts from the dorsal neural tube and surface ectoderm, and bone morphogenic proteins (BMPs) from the overlying ectoderm result in the downregulation of somitic marker Pax3 and the expression of MRFs [17, 18].

Upstream of Myf5 and MyoD, the expression of homeobox transcription factors Pax3 and Meox1 commences early during somite development and becomes restricted to the dorsal somite during specification of the dermomyotome [19, 20]. While Pax3 is both necessary and sufficient to induce skeletal myogenesis, Meox1 is required for *Pax3* expression and subsequent myogenic differentiation [21–23].

## Roles of nuclear receptors in cellular function

The retinoid acid receptors (RARs), namely RARα, RARβ and RARγ, belong to the family of class II nuclear receptors, which bind to DNA constitutively as heterodimers with the retinoid X receptors (RXRs). Unliganded RAR–RXR heterodimers bound to RA response elements (RAREs) associate with corepressor protein complexes and prevent transcription at target promoters [24]. To activate transcription of a RA target gene, binding of RA to RAR produces a conformational change in the structure of RAR that allows dissociation of the corepressor complex and recruitment of a coactivator complex [25]. Within the RAR–RXR heterodimer, activation of RXR alone is non-permissive since it is not generally sufficient to induce gene expression [26, 27]. However, RAR–RXR heterodimers bound by ligands for both receptors may allow enhanced expression of their targets, demonstrating synergistic activation potential between RAR and RXR [28].

**Fig. 2:**
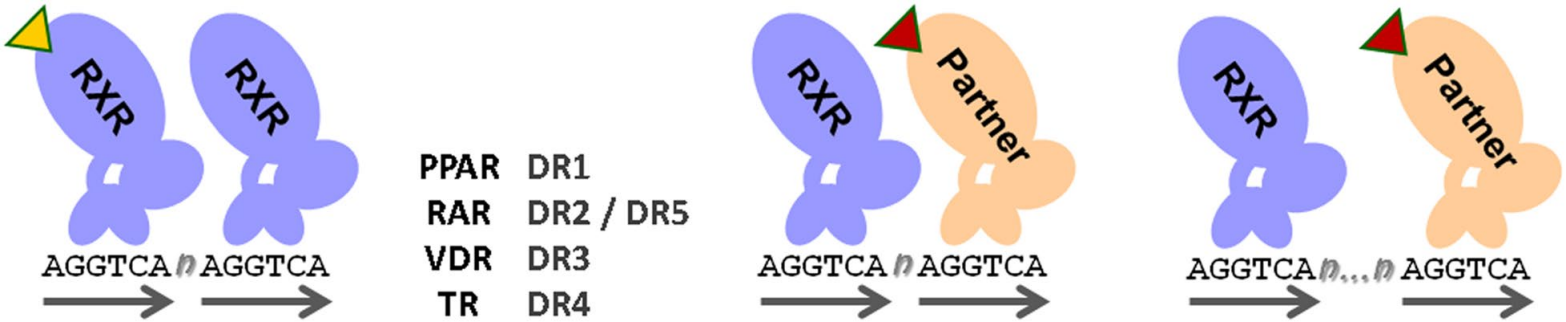
Schematic representation of the dimerization of RXR with nuclear receptor partners, such as the peroxisome proliferator-activated receptor (PPAR), RAR, the vitamin D receptor (VDR), and the thyroid hormone receptor (TR), and the number of spacer nucleotides between the two direct repeats of canonical DNA binding sites (DRn)

There are three subtypes of RXR, namely RXRα, RXRβ and RXRγ and a remarkable feature of these RXRs is that they bind to their DNA motifs constitutively as homodimers, or as heterodimers with many other nuclear receptors [29–31]. Consequently, the roles of RXRs in target gene transcription is affected by the type of dimerization and the number of spacer nucleotides between the two direct repeats of canonical DNA binding site (Fig. 2) [32, 33]. Nonetheless, the association of RXR with nuclear receptor partners can change during cellular differentiation [34, 35]. Interestingly, pluripotent embryonal carcinoma (EC) cells containing a dominant negative RARα that blocks the DNA binding capacity of the receptor [36, 37], are not able to commit to the skeletal muscle lineage but can undergo neuronal differentiation following RA induction [38, 39]. However, RXR, but not RAR, is essential for the differentiation of skeletal myoblasts [40]. Moreover, knockdown of RXRα attenuates rexinoid-promoted myoblast differentiation and fusion [41]. Although the precise role of RXR in myogenic differentiation remains to be determined, advances in next generation sequencing have allowed the mapping of RXRα binding sites genome-wide in other cellular systems [34, 42, 43].

## Bexarotene promotes myogenic differentiation of ES cells following mesoderm specification

Early studies found that the differentiation of pluripotent cells in vitro can be directed towards skeletal myogenesis using low concentrations of RA [5, 6]. However, treating embryonic stem (ES) cells during embryoid body (EB) formation with bexarotene leads to an increase in the rate of myogenic differentiation over treatment with RA, from approximately 3–16% [10]. To identify genes that are preferentially targeted by bexarotene compared to RA during the differentiation of ES cells into skeletal myocytes, gene expression profiles have been generated using Affymetrix microarrays (GSE94779). While bexarotene and RA regulate similar genetic pathways, the expression of certain stage-specific transcription factors is preferentially enhanced in bexarotene-treated ES cells. Thus, bexarotene may promote skeletal myogenesis in ES cells through early expression of transcription factors and signaling molecules that are generally required for myogenic specification in the embryonic somite.

**Fig. 3:**
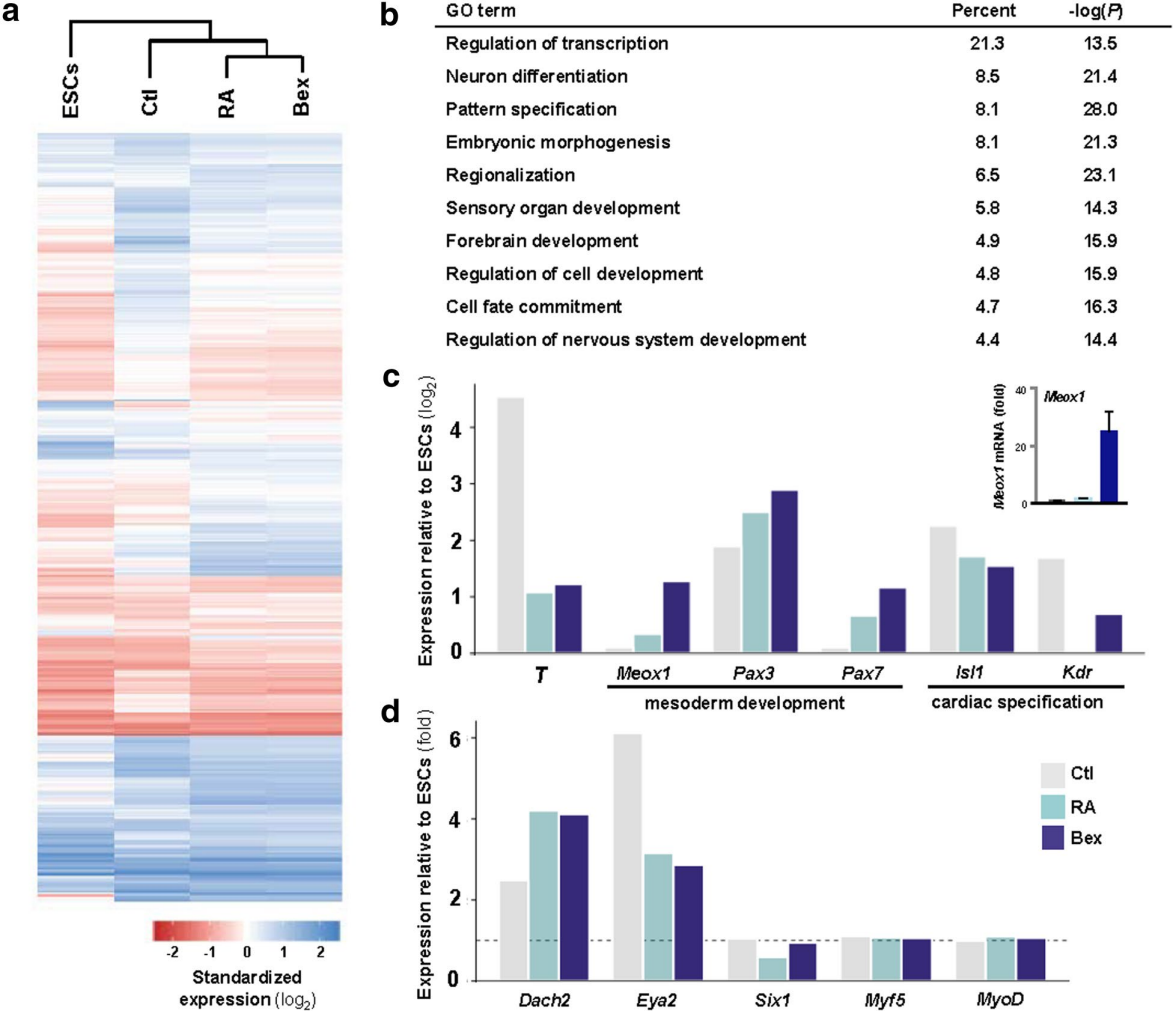
Gene expression profiles in ES cells differentiated with bexarotene or RA. **a** Mouse ES cells (*D3 line*) were grown as hanging drops for 2 days and then in suspension for an additional 5 days, to allow the formation of EBs in the presence or absence of bexarotene (Bex, 50 nM) or RA (10 nM). Undifferentiated ES cells were analyzed in parallel (ESCs). Total mRNAs isolated from the cells were subjected to Affymetrix microarray analysis. Primary analysis and quality control was performed using the Affymetrix Expression Console (version 1.1.2637.26569). Probe intensity data was normalized using robust multi-array average (RMA), and a threshold of ±1.5-fold was used to define differential expressed between each condition. Euclidean distance-based clustering was used to visualize the expression of differentially expressed genes. Raw and processed data are deposited in NCBI’s Gene Expression Omnibus (GEO) database, under accession number GSE94779. Standardized values of expression (log_2_) for genes that showed a difference in expression greater than ±1.5-fold between bexarotene-treated EBs and untreated EBs (Ctl). **b** GO terms associated with genes differentially expressed following bexarotene treatment found using the DAVID. **c** Expression of selected genetic markers for mesoderm specification (*T*), mesoderm development (*Meox1*, *Pax3*, *Pax7*) and cardiac specification (*Isl1*, *Kdr*). Values are given as difference in expression (log_2_) between the EBs and ESCs. Inset is the relative mRNA levels of *Meox1* validated by qRT-qPCR analysis and plotted as fold change relative to untreated EBs after being normalized to GAPDH. **d** Expression of early myogenic transcription factors given as the fold change relative to ESCs. Values in ESCs are indicated by a* dashed line*

Specifically, 1,038 probe sets for 924 genes exhibit expression greater than ±1.5-fold in bexarotene-treated EBs relative to untreated EB control (Fig. 3a). Among the bexarotene responsive genes, 380 genes are upregulated while 544 genes are downregulated. Functional annotations using the Database for Annotation, Visualization and Integrated Discovery (DAVID) and gene ontology (GO) [44, 45] reveal that 21% of bexarotene responsive genes are involved in the regulation of transcription, the highest populated GO category identified (Fig. 3b). Among others, associations with pattern specification and neuron differentiation are also found. Interestingly, each GO category contains an approximately equal number of up- and down-regulated genes, reflective of the diverse changes in gene expression that occur prior to lineage commitment and tissue-specific gene expression at the early stages of tissue specification. Thus, GO analysis demonstrates that bexarotene allows coordinated regulation of developmental factors during the myogenic differentiation of ES cells.

Expression of the mesodermal marker *brachyury T*, which is induced in ES cells through cellular aggregation [46], is found in both treated and untreated EBs (Fig. 3c). In the absence of ligand, *brachyury T* is upregulated by more than 16-fold in the EBs in comparison to the ES cells. However, its expression is notably reduced by bexarotene relative to the untreated EB control. Downregulation of *brachyury T* reflects a facilitated progression of differentiation in bexarotene-treated EBs, in contrast to control EBs which retain a mesodermal progenitor phenotype. More importantly, bexarotene also enhances the expression of *Meox1* and *Pax3* (Fig. 3c). While not important for embryonic myogenesis, the *Pax3* paralog *Pax7* is required for adult myogenesis and muscle regeneration [47] and its expression is also upregulated by bexarotene. These results suggest that the temporal regulation of transcription factors in bexarotene-treated EBs mirrors the pattern of transcription factor expression observed during in vivo somitogenesis and early myogenesis, implying regenerative potential.

In addition to the Pax genes and the MRFs, the transcription factors Dach2, Eya2 and Six1 have been shown to play important roles in embryonic myogenesis [48–50]. In particular, interactions between Dach2 and Eya2 and between Eya2 and Six1 synergistically regulate the transcription of muscle-specific genes [48]. While the expression of neither *Myf5* nor *MyoD* is induced in bexarotene-treated EBs in early differentiation, *Dach2* expression which shows spatial and temporal similarity to *Pax3* expression during somite development [48], is upregulated and *Eya2* expression is downregulated relative to control EBs (Fig. 3d).

While enhancing skeletal myogenesis in pluripotent stem cells, both bexarotene and RA inhibit cardiomyogenesis and the expression of *Gata4* [11, 51]. Bexarotene and RA downregulate the expression of *Isl1* and *Kdr* (also known as *Flk1),* two genetic markers of precardiac mesoderm (Fig. 3c) [52, 53]. During embryogenesis, specification of precardiac mesoderm occurs in a population of cells located in the embryonic primitive streak prior to the specification of myogenic progenitors in the somite [53]. Taken together, these data suggest that bexarotene and RA inhibit the specification of cardiac progenitors and therefore prevent the differentiation of ES cells into cardiomyocytes.

## Specification of paraxial mesoderm by bexarotene precedes its specification by RA

**Fig. 4:**
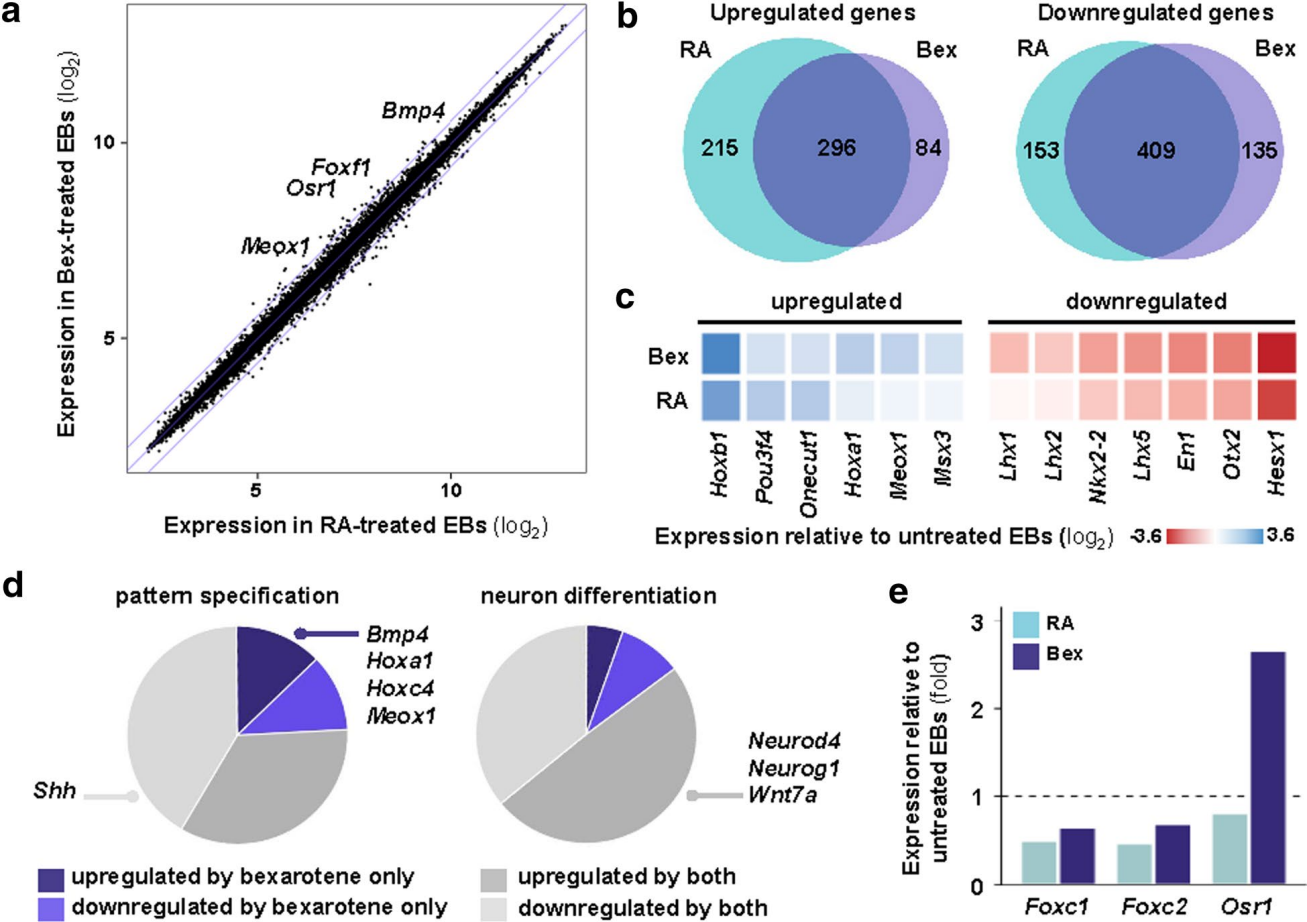
Bexarotene modulates RA-dependent and -independent gene expression. **a** Global gene expression in bexarotene- or RA-treated EBs (log_2_).* Outer lines* mark ±1.5-fold differences in expression between the two treatments. **b** Overlap in up- or down-regulated genes between EBs treated with bexarotene or RA. **c** Expression of selected homeobox genes showing differential expression between bexarotene and RA. **d** Bexarotene-regulated genes involved in pattern specification and neuron differentiation categorized according to their similarity of expression in RA-treated EBs. **e** Difference in expression from untreated EBs of transcription factors involved in intermediate mesoderm specification. Values in untreated EBs are indicated by a *dashed line*

Given the fact that both RA and bexarotene are ligands of nuclear receptors [10], it is not surprising that few genes showed a greater than 1.5-fold difference in expression between ES cells treated with bexarotene and those treated with RA (Fig. 4a). The majority of genes that are differentially expressed in bexarotene-treated EBs are similarly regulated by RA, indicating that bexarotene and RA may target similar molecular pathways (Fig. 4b). 78% of genes upregulated by bexarotene with respect to untreated EB control are also upregulated by RA. Similarly, 75% of genes downregulated by bexarotene are also downregulated by RA. Few genes that are upregulated by both ligands show a difference in expression greater than 1.5-fold between bexarotene- and RA-treated EBs, suggesting that nuclear receptor activation and the effect of bexarotene on transcription of RA targets are gene- or promoter-dependent. For example, several homeobox genes are differentially expressed between bexarotene- and RA-treated EBs (Fig. 4c). This set of genes included the Hox genes *Hoxa1* and *Hoxb1,* which both have well annotated RAREs upstream of their promoters [54, 55] and which are both enhanced by bexarotene.

EBs treated with bexarotene or RA show a more than two-fold decrease in *brachyury T* expression relative to untreated EBs, reflecting the timely progression of differentiation in both bexarotene- and RA-treated cells. *Brachyury T* is similarly expressed in both treated conditions, suggesting that the divergence in efficiency between bexarotene and RA in promoting skeletal myogenesis in EBs occurs after mesoderm specification (Fig. 3c). Notably, the expression of *Pax3* and *Meox1* is greater in EBs treated with bexarotene than treated with RA. While *Pax3* is upregulated in RA-treated and untreated EBs, the latter reflecting a non-specific role in mesodermal tissue specification, *Meox1* expression is induced exclusively in bexarotene-treated EBs (Fig. 4d). In addition, neither the expression of *Myf5* nor *MyoD* is upregulated at this stage of differentiation, suggesting that bexarotene enhances skeletal myogenesis at the early stages of myotome specification.

Coupled with the specification of paraxial and somitic mesoderm, demonstrated by activation of *Pax3* and *Meox1,* is the downregulation of the intermediate mesoderm marker *Osr1* [56]. In chick embryos, compound null mutants for the transcription factors Foxc1 and Foxc2, which are highly expressed in paraxial mesoderm, lack somites and the expression of *Osr1* expands medially, suggesting that Foxc1 and Foxc2 are required for correct mesoderm specification during embryonic development through inhibition of *Osr1* expression [57]. However, while the expression of *Foxc1* and *Foxc2* is repressed by both treatments, *Osr1* is highly expressed in EBs treated with bexarotene, and its expression was threefold greater than in RA-treated EBs (Fig. 4e). Given the multifunctional nature of RXR, these results suggest that bexarotene also regulates the expression of genes independent of RA signaling, which may contribute to the enhanced myogenic phenotype observed in these cells.

## Induction of BMP signaling by bexarotene may promote myogenic specification

**Fig. 5:**
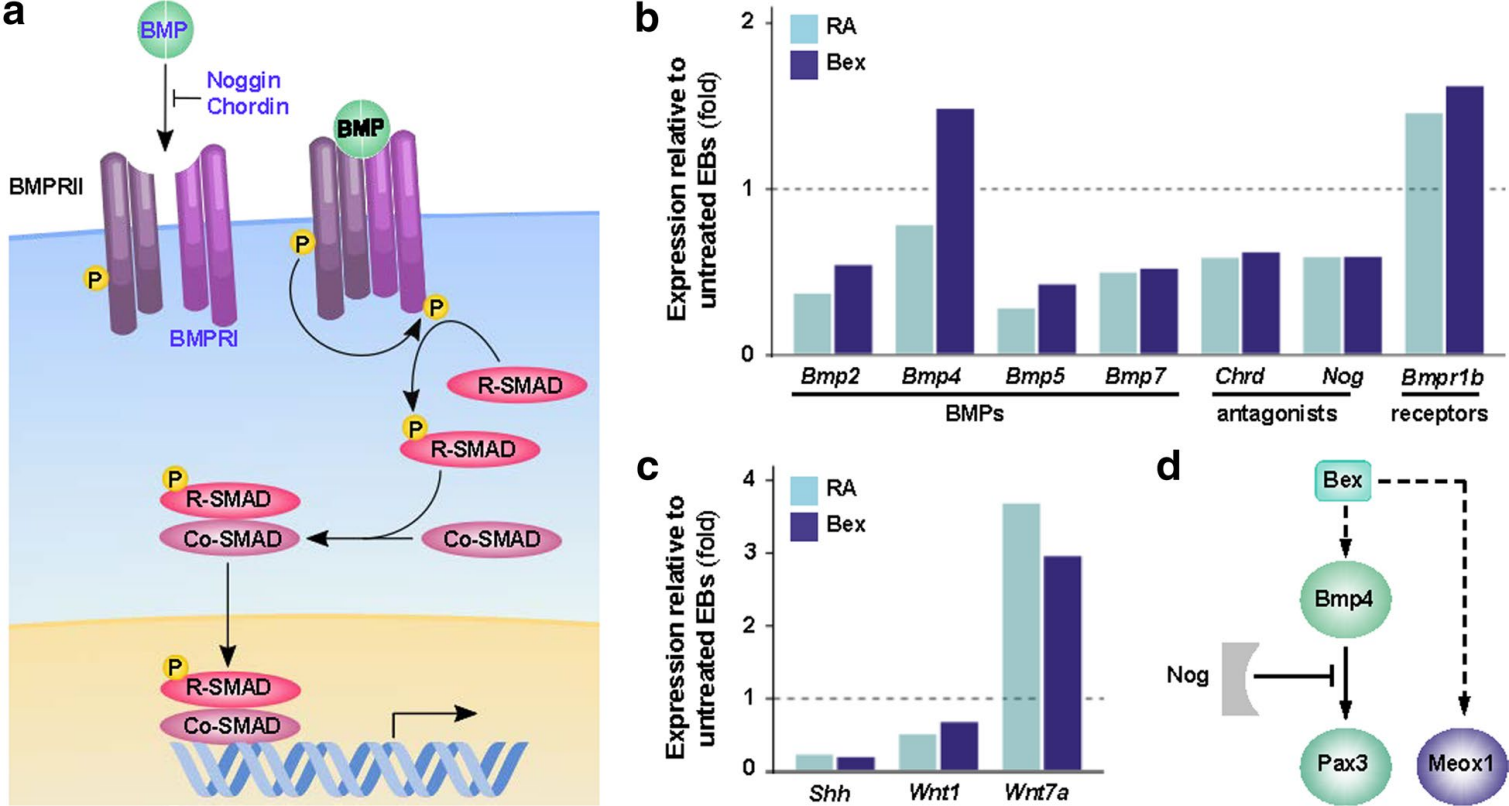
Early activation of BMP signaling may mediate bexarotene-promoted myogenic specification. **a** Schematic of BMP signaling. Factors differentially regulated by bexarotene are* texted in blue*. **b** Members of BMP pathway were differentially expressed in bexarotene- or RA-treated EBs. **c** Shh and Wnts differentially expressed in bexarotene- and RA-treated EBs. Values are given as a fold change relative to their corresponding values in untreated EBs that are indicated by a *dashed line*. **d** Representational diagram of the inferred influence of bexarotene in the enhancement of myogenic specification

During embryogenesis, paracrine signaling is critical for correct patterning and morphogenesis. In particular, the expression of *Myf5* and *MyoD* in the dermomyotome is regulated by coordinated regulation of Shh, Wnt and BMP signaling [17, 18]. Nevertheless both bexarotene and RA promote coordinated regulation of secreted and intermediate signaling factors normally required for myogenic specification and differentiation in vivo (Fig. 5).

With multiple roles during development including cell growth and differentiation, BMPs signal through kinase transmembrane receptors and the SMAD family of transcription factors [58, 59]. BMP signaling is required for correct temporal regulation of MRF expression, and it has been shown to inhibit myogenic differentiation and increase the number of myogenic progenitors by promoting *Pax3* expression [17]. Interestingly, several genes encoding members of the BMP signaling pathway are differentially expressed between bexarotene- and RA-treated EBs (as shown in blue in Fig. 5a). Specifically, the expression of *Bmp4* is about twofold greater in EBs treated with bexarotene than with RA (Fig. 5b). In comparison to untreated EB control, RA downregulates the expression of BMP-2, -4, -5 and -7, while bexarotene downregulates the expression of BMP-2, -5 and -7 only (Fig. 5b).

Additionally, progression into differentiation is regulated by BMP antagonists, which may bind directly to BMPs and prevent activation of BMP receptors. The antagonist noggin *(Nog)* and chordin *(Chrd)* do not show a notable difference in expression between bexarotene and RA treatments, but are downregulated relative to EB control by bexarotene and RA. Moreover, both bexarotene and RA enhance the expression of the BMP receptor *Bmpr1b.* These data may reflect early induction of BMP signaling by bexarotene, particularly in the expression of *Bmp4*, leading to an increase in the number of *Pax3*-positive myogenic progenitors.

A positive regulator of *Myf5, Shh* expression is strongly repressed by RA and bexarotene (Fig. 5c). *Wnt1*, which is a strong activator of *Myf5* expression through the transcription coactivator β-catenin and the TCF/LEF family of transcription factors [18], is downregulated from the EB control by RA. Contrarily, *Wnt7a* which activates the expression of *MyoD* independent of β-catenin [18], is induced by both bexarotene and RA. Embryonic somites exposed to Shh are preconditioned to the induction of myogenesis via Wnt signaling by the dorsal neural tube and surface ectoderm [60]. Coupled with the inhibition of BMP signaling, myogenic progenitors become committed to the skeletal muscle lineage and are capable of undergoing temporal differentiation into skeletal myocytes. Therefore, the coordination of intercellular signaling observed during embryonic myogenesis may be preserved and perhaps promoted in bexarotene- and RA-treated EBs.

## Implication of rexinoid signaling in myogenic specification

Bexarotene, a RXR agonist, effectively enhances skeletal myogenesis in ES cells with a greater efficacy than RA [10]. In addition to targeting genes expressed at the early stages of myogenic specification, bexarotene promotes early expression of signaling factors required to expand the myogenic progenitor cell pool and ultimately lead to an efficient generation of skeletal myocytes.

Interestingly, both bexarotene and RA cannot bypass the inhibition of myogenesis resulting from the functional loss of β-catenin, as neither could enhance the expression of *MyoD* nor induce skeletal myogenesis in stem cells expressing dominant negative β-catenin [10, 61]. During the specification of myogenic progenitors in the dorsal somite, canonical and noncanonical Wnt signaling act in parallel to regulate the expression of *Myf5* and *MyoD* in distinct populations of cells [18]. Wnt1 which strongly induces *Myf5* expression via β-catenin is repressed by bexarotene or RA (Fig. 4). However, expression of *Wnt7a* is induced by bexarotene and RA (Fig. 5), indicating that the activation of *MyoD* in β-catenin loss-of-function stem cells depends on additional factors. Since *Myf5* is regulated by canonical Wnt signaling and lies genetically upstream of *MyoD*, it is possible that loss of *Myf5* induction precedes repression of *MyoD* in cells expressing a nonfunctional β-catenin. This suggests that myogenesis is promoted by bexarotene and RA in ES cells in part through coordination of paracrine signaling, therefore allowing similar temporal control of lineage-specific transcription factors as observed during embryonic development.

Classical models of RA signaling suggest that RXR functions as a silent or non-permissive partner to RAR, where activation of RAR–RXR heterodimers is dependent on RAR ligand. In the presence of a RXR ligand, the expression of RA targets may be enhanced however, resulting from weaker association with corepressors and/or stronger association with coactivators. However, the induction of several genes, including *Hoxa1* and *Meox1*, only occurred in the presence of bexarotene. At the *Hoxa1* locus, functional RAREs are required to activate its expression in order to specify anterior tissue along the AP axis of the elongating embryo [52]. As EBs undergo ligand-induced myogenesis, it may be possible that RA-mediated regulation of *Hoxa1* is blocked, but that the locus remains responsive to RXR activation. In a different manner, *Meox1* expression may be activated by rexinoids as a result of early induction of the myogenic program. These observations indicate diverse transcriptional pathways regulated by rexinoids in ES cells, while also demonstrating the rexinoid’s potential for application in a variety of locus-specific contexts.

Nuclear receptors, particularly those existing in RXR heterodimers, are susceptible to multiple signals that synergistically modulate the activation of transcription at target gene promoters. Binding of RAR or RXR agonist to RAR–RXR causes a conformational change in the dimer, altering the activation potential of the partner receptor and the resulting level of transcription at genetic targets. As the formation of EBs may induce the synthesis of endogenous RA, rexinoids may regulate the expression of RA target genes in part through synergistic relationships within the RAR–RXR heterodimer. However, bexarotene enhances skeletal myogenesis in EC stem cells co-treated with a RAR antagonist and in cells expressing dominant negative RAR, indicating that rexinoids can enhance myogenesis through a pathway independent from functional RAR [10]. In addition, expression profiling reveals that several genes are regulated differently by RA and bexarotene, but few whose expression is naturally regulated by RA shows an enhanced phenotype with bexarotene. Thus endogenous RA may not be sufficient to allow nuclear receptor synergy between RAR and RXR at many loci in differentiating EBs.

The displacement of repressive protein complexes, such as SUZ12, is affected by the specific RAR isotype [62]. The transcription of RA target genes also depends on the ability of RAR–RXR to recruit coactivators, and the type of coregulator bound to RAR–RXR, such as pCIP, p160 or p300, affects the activation of target genes [25]. DNA-binding by RAR is cell type-specific, and most binding sites are occupied in the absence of RA [63]. Furthermore, the influence of RA is promoter-specific, suggesting diverse transcriptional potential of RARs even within specific cell types [64]. Therefore, in addition to synergistic relationships between RAR and RXR activation, the isotype of receptors, the strength of coregulator association and locus-specific promoter affect the transcriptional activity of RA target genes.

Due to the multifunctional nature of RXR, the application of rexinoids in the in vitro generation of muscle tissue or in muscle regeneration may be achieved through multiple molecular pathways. Future work to determine the potential of utilizing the regulation of nuclear receptor signaling in these fields and the optimal treatment regimes for rexinoid-related therapies will be required.

## Abbreviations

Chrd: chordin
EC: embryonic carcinoma
ES: embryonic ste
bHLH: basic helix-loop-helix
BMP: bone morphogenic proteins
GO: gene ontology
MRF: myogenic regulatory factors
Nog: antagonist noggin
PPAR: peroxisome proliferator-activated receptor
RAR: retinoid acid receptor
RXR: retinoid X recepto
Shh: sonic hedgehog
TR: thyroid hormone receptor
VDR: vitamin D receptor
